# Hypothesis of “stroke-stop” formula: a tool for risk index determination in development of acute cerebrovascular disease in asymptomatic individuals with carotid stenosis

**DOI:** 10.1186/s12883-021-02337-y

**Published:** 2021-08-11

**Authors:** Іvan Kopolovets, Peter Berek, Peter Stefanic, Dmytro Lotnyk, Rastislav Mucha, Zdenka Hertelyova, Stefan Toth, Nadiya Boyko, Vladimir Sihotsky

**Affiliations:** 1 Clinic of Vascular Surgery, Eastern Slovak Institute of Cardiovascular Diseases and Faculty of Medicine, Pavol Jozef Safarik University, Ondavska 8, 04011 Kosice, Slovak Republic; 2 Uzhhorod National University, Research Development and Educational Center of Molecular Microbiology and Mucosal Immunology, Uzhhorod, Ukraine; 3 Department of Physics Cornell University Clark Hall, New York, USA; 4 Institute of Neurobiology, Biomedical Research Center of the Slovak Academy of Sciences, Kosice, Slovak Republic; 5 Institute of Experimental Medicine, Faculty of Medicine, Pavol Jozef Safarik University, Kosice, Slovak Republic; 6 Clinic of Cardiology, Eastern Slovak Institute of Cardiovascular Diseases and Faculty of Medicine, Pavol Jozef Safarik University, Kosice, Slovak Republic

**Keywords:** Atherosclerosis, Carotid artery stenosis, Vulnerable plaque, Vascular markers, Stroke, Personalized medicine

## Abstract

**Background:**

Extracranial carotid artery disease is considered a risk factor for developing acute cerebrovascular diseases. The paper suggests the “Stroke-Stop” formula as hypothesis for the determination of the risk of developing stroke in asymptomatic individuals with carotid stenosis. The formula is based on a mathematical calculation of the major risk factors for stroke: the degree of ICA (internal carotid artery) stenosis, the morphological structure of the atherosclerotic plaque and the level of lipoprotein-associated phospholipase A2 (Lp-PLA2) concentration.

**Methods:**

The cross sectional study included 70 patients with atherosclerotic ICA stenosis. Among vascular inflammatory markers, Lp-PLA2 was determined with concentration 252.7–328.6 mg/l. The obtained results were evaluated using descriptive statistics (the frequency, percentage ratio) as well as the one-way analysis of variance (ANOVA) and chi-square test.

**Results:**

The risk of stroke development is eminently increasing with the progression of ICA stenosis and elevation of Lp-PLA2 levels. In patients with echolucent plaque, the risk of stroke development was significantly higher in correlation with patients with echogenic plaque. Based on calculations using “Stroke-Stop” formula, three main groups were generated: low (< 70 points), medium (70–100 points) and high (> 100 points) risk of stroke development.

**Conclusions:**

Hypothesis of “Stroke-Stop” formula is proposed for better selection of patients who should be indicated for surgical treatment and will be evaluated in prospective study. In order to verify this hypothesis, we plan to do prospective study using “Stroke-Stop” formula for ipsilateral annual stroke rate in asymptomatic individuals with carotid stenosis who receive conservative therapy.

## Highlights

- Stenosis, ulceration of the atherosclerotic plaque and Lp-PLA2 concentration are calculated
- 3 groups of stroke risk development were set (low, medium and high)
- Proposed formula form personalized approach to the diagnosis and prevention of stroke

## Background

Extracranial carotid artery disease is considered a risk factor for developing acute cerebrovascular diseases [1]. Despite a large number of scientific researches dedicated to prevention, diagnosis and treatment of stroke, the problem is still relevant [2]. The ratio of ischemic: hemorrhagic stroke incidence is (80–85%):(15–20%), respectively and the leading cause of cerebral ischemia (approximately 40–45%) is atherosclerotic internal carotid artery (ICA) stenosis [3]. Due to timely diagnosis and administration of conservative treatment (antiplatelet therapy), over the last 3–4 decades the rate of stroke in asymptomatic and symptomatic individuals with carotid stenosis has fallen significantly [4, 5]. Despite this trend, a lot of individuals with carotid stenosis receive endovascular or surgical treatment (CAS/CEA) [5, 6].

The process of stroke occurrence in individuals with carotid stenosis is influenced by the degree of ICA stenosis and the clinical course. For example, the annual rate of stroke in the NASCET trial with 70–99% stenosis (using outcomes at 2 years), was 13% and approximately 7%/year for 50–69% stenosis [7].

The average annual rate of ipsilateral stroke in asymptomatic individuals with ICA stenosis > 70% is 1–3%, while in patients with at least 50% stenosis, it approximates 0.8% [8].

To determine the treatment strategy for patients with carotid stenosis, there have been conducted randomized trials that demonstrated the advantages of surgical methods of treatment (СEA/CAS) over conservative ones, primarily in symptomatic individuals with ICA stenosis [3, 9]. Using pooled results from the NASCET, the ECST and the VA309 Study [10], patients with 70–99% stenosis (NASCET) and without near occlusion who had CEA and medical intervention had an overall reduction in the rate of any peri-operative stroke or death or subsequent ipsilateral stroke of 3.2%/year as compared to patients who had medical intervention alone. This was a 16% total reduction in event rate, from approximately 25% at 5 years after recruitment [10].

In case of symptomatic individuals with ICA stenosis, there have been a clear advantage of surgical preventing ischemic stroke, while among asymptomatic individuals with ICA, such advantage has not been observed [11, 12].

Therefore, the criteria for selecting patients for surgical prevention of acute cerebrovascular disease among asymptomatic individuals with carotid stenosis are broadly discussed nowadays [13, 14]. There have been several studies, where in addition to atherosclerotic ICA stenosis, the morphological structure of the atherosclerotic plaque was taken into consideration [15–18]. In recent years, researchers have widely investigated vascular inflammatory markers as well as the relationship between their concentration and the risk of developing atherosclerotic complications [19]. Specific biomarkers are crucial tool since enabling the early prevention of non-symptomatic stages of diseases and also support prognostic/predicted and patient- centered treatment. The useful biomarker has to correlate with clinical parameters, such as specific symptoms, clinical signs and validated diagnostic tests. One of the inflammatory markers correlating to atherosclerosis complications with high specificity is the lipoprotein-associated phospholipase A2 (Lp-PLA2) [20, 21].

Lp-PLA2 expression was significantly higher in plaques of symptomatic patients than asymptomatic patients (1.66+/− 0.19 versus 1.14+/− 0.10, *P* < 0.05) and localized mainly to shoulder and necrotic lipid core areas in colocalization with oxLDL and macrophage content [22].

The meta-analysis, which studied the association between baseline levels of Lp-PLA2 activity/mass and stroke risk, showed that elevated Lp-PLA2 levels are associated with higher stroke risk. Lp-PLA2 levels can be considered as factor to predict stroke in high-risk individuals [23].

Elkind et al. studied Lp-PLA2 mass and hs-CRP in 467 patients after a first ischemic stroke and concluded that Lp-PLA2, but not hs-CRP, was associated with the recurrence of ischemic neurological events [24].

Duplex ultrasonography is one of the main screening methods for detection of atherosclerotic ICA stenosis [25, 26]. It allows detecting the degree of ICA stenosis as well as evaluating the structure of the atherosclerotic plaque [27]. It is the degree of stenosis that serves as the main indication for performing carotid endarterectomy [5]. However, according to recent reports, approximately 20–25% of symptomatic patients have ICA stenosis < 70% [28]. This means that one criterion alone, namely the degree of ICA stenosis is not enough to determine the risk of stroke development in asymptomatic individuals since this category of patients (20–25%) is not under active supervision by physicians.

Together, these data confirm that the problem of early detection of patients at the highest risk of developing stroke remains relevant.

## Methods

### Patient selection

This cross-sectional study has been cleared by the Ethics Committee of the East Slovak Institute of Cardiovascular Diseases (07/12/2015) for human studies and that patients have signed an informed consent.

The study included 70 patients with atherosclerotic ICA stenosis (symptomatic individuals with ICA stenosis > 50%, and asymptomatic individuals with ICA stenosis > 70%), where 44 (63%) patients were males, and 26 (37%) patients were females who were hospitalized and subjected to carotid endarterectomy at the Clinic of Vascular Surgery of the Eastern Slovak Institute of Cardiovascular Diseases and the Faculty of Medicine of Pavol Jozef Safarik University, Kosice.

Symptomatic patients were defined as patients who had suffered an ischemic stroke or transient ischemic attack (TIA) within last 6 months, while asymptomatic patients were defined as those who did not suffer any stroke or TIA within last 6 months. All patients were examined by neurologist, who identified carotid stenosis as the most probable cause for neurological symptomatology.

Depending on the clinical course, the patients were divided into two groups:

- Group I included 30 (43% of all 70 tested) symptomatic individuals with carotid stenosis; among them, 20 (66.6% of Group I) patients had a history of ischemic stroke, and 10 (33.3% of Group I) patients had a history of transient ischemic attack (TIA);
- Group II included 40 (57% of all 70 tested) asymptomatic individuals with carotid stenosis.

Exclusion criteria were severe neurologic impairment (hemiplegia, coma), recent heart failure, uncontrolled hypertension, atrial fibrillation, critical limb ischemia, acute and chronic liver disease, acute and chronic renal failure, decompensated metabolic disease, acute and chronic infection, patients with history of cancer and autoimmune disease.

The control group included 20 (10 men and 10 women) individuals; without any statistically significant difference in age and sex. The average age was 38 ± 5.2 years, and the group consisted of individuals without any sign of atherosclerotic disease and without stenosis of carotid arteries on ultrasound examination.

The study characteristics of the current participants (excluding negative controls) are presented in the Table 1.

**Table 1:** Characteristics of the study groups

	Total	Group I (sympt. ***n*** = 30)	Statistical importance	***P***-value	Group I (asympt. ***n*** = 40)	Statistical importance	P-value
Male	43 (61.4%)	22 (73.3%)	N/I	0.6	21 (26.7%)	N/I	0.6
Female	27 (38.6%)	16 (53.3%)	N/I	0.4	11 (46.7%)	N/I	0.4
Current smoking	29 (41.4%)	14 (46.7%)	N/I	0.8	15 (37.5%)	N/I	0.8
Ischemic heart disease	48 (68.5%)	22 (73.3%)	N/I	0.7	26 (65.0%)	N/I	0.9
Hypertension	65 (92.9%)	28 (93.3%)	N/I	1	37 (92.5%)	N/I	1
Diabetes mellitus	17 (24.3%)	8 (26.7%)	N/I	0.8	9 (22.5%)	N/I	0.9
Peripheral artery disease	3 (4.3%)	2 (6.7%)	N/I	0.6	1 (2.5%)	N/I	0.6

*N/I* not important, *sympt.* symptomatic individuals with carotid stenosis, *asympt.* asymptomatic individuals with carotid stenosis

### Preoperative ultrasound imaging of the atherosclerotic plaque

The degree of ICA stenosis, as well as the morphological structure of the atherosclerotic plaque, was determined in the preoperative period using ultrasonography. Duplex scanning of the carotid arteries was performed using the Philips HD11XE ultrasound machine with an 8 MHz linear transducer. The degree of stenosis was evaluated according to the “Consensus Panel Gray-Scale and Doppler US Criteria for Diagnosis of ICA Stenosis” through the determination of the peak systolic velocity (PSV) and the end diastolic velocity (EDV) within the ICA [29].

The structure and composition of the atherosclerotic plaque was assessed using echogenicity: echolucent, heterogeneous and echogenic carotid plaques.

Echolucent and heterogeneous plaques were assessed as “unstable” plaques; echogenic plaques were assessed as “stable” plaques [26].

### Marker of inflammation in patients with carotid artery stenosis

Blood samples of all patients were taken 1 day before the surgery, and the concentration of specific markers, namely, Hpx, Lp-PLA2, and IL-4, were determined. The levels of these parameters were determined using the ELISA method (human lipoprotein associated phospholipase A2, Cussabio, USA; Human Hpx, Abcam, UK), and the results were evaluated with the Tukey test. The plasma level of IL-4 was measured using an ELISA kit Human IL-4 Platinum ELISA (eBioscience, San Diego, USA). Blood samples for the measurement of specific markers were centrifuged after collection at 2.500 rpm for 10 min. Serum samples were then frozen at − 80 °C until analysis. Samples were processed using Synergy H4 multiplate reader (BioTek Vermont, USA).

### Procedure of carotid endarterectomy

All patients underwent eversion carotid endarterectomy in general anesthesia. We used transcranial cerebral oximetry as cerebral monitoring during general anesthesia (device INVOS™ 5100 C, Somanetics, Troy, MI, U.S.A.). All patients were prescribed with antiplatelet therapy postoperatively and were evaluated by an independent neurologist after the operation.

### Histopathology

In the postoperative period, histological assessment of the atherosclerotic plaque removed during carotid endarterectomy was performed. The selected atherosclerotic plaque was fixed in a 4% solution of neutral formalin; then, decalcification was carried out, and the material was poured in paraffin blocks 1 cm in size. 5-μm-thick sections of paraffin blocks cut by a microtome were studied histologically. The blocks were examined in the longitudinal section, in the most stenotic area. The hematoxylin and eosin staining were used. Histopathological characteristics of the retrieved carotid plaques were reported according to the updated American Heart Association classification of advanced atherosclerosis and a previously well-validated scoring system published by Lovett et al. [17]. For each plaque, the following features were recorded: the rupture of the fibrous cap, lipid core size, nodular calcification, neovascularisation, inflammatory infiltrate, infiltration of the fibrous cap, proportions of fibrous tissue, intraplaque hemorrhage, presence of foam cells, and surface thrombus. Based on the presence of these features in each plaque, an overall stability rating was given by the histopathologist as “unstable” or “stable.” Unstable plaques demonstrated many or all features, and “stable” plaques demonstrated none of them [30–32].

### Statistical methods

The obtained results were evaluated using descriptive statistics (the frequency, percentage ratio) as well as the one-way analysis of variance (ANOVA) (Minitab Inc. version 11.24, Coventry, UK) and chi-square test (Preacher, K. J. (2001, April). Calculation for the chi-square test: An interactive calculation tool for chi-square tests of goodness of fit and independence). The relationship between two variables – the group of symptomatic individuals and the group of asymptomatic ones – was evaluated using the one-way analysis of variance (ANOVA) (Minitab Inc. version 11.24, Coventry, UK). The correlative relationship was evaluated using Pearson’s correlation coefficient (Pearson correlation test, MINITAB Inc., Coventry, United Kingdom). The relationship between more than two variables (symptomatic patients, asymptomatic patients, and the control group) was evaluated using the Tukey’s HSD test (Minitab Inc. version 11.24, Coventry, UK); the data were considered statistically significant at *p* < 0.05.

### Theory

This study aims to analyze diagnostic criteria for carotid stenosis in the patients presented. The parameters under study (the degree of ICA stenosis, the morphological structure of the atherosclerotic plaque and the concentration of Lp-PLA2) were analyzed and compared between the two groups. Based on the results obtained, the modelling of the studied parameters was carried out, which is illustrated in the hypothesis of “Stroke-Stop” formula.

## Results

The average patients’ age was 69 ± 7.5 years. There was no statistically significant difference in age and sex between the groups (Group I - symptomatic individuals with carotid stenosis, Group II - asymptomatic individuals with carotid stenosis.

### Results of ultrasound examination

The assessment of the atherosclerotic plaque echogenicity according to the ultrasonographic data has revealed that echolucent atherosclerotic plaque was observed in 19 (27.1%) patients, a heterogeneous atherosclerotic plaque was detected in 29 (41.4%) patients and a echogenic atherosclerotic plaque was found in 22 (31.5%) patients. The ratio of the atherosclerotic plaque echogenicity between Group I and Group II is presented in Table 2.

**Table 2:** Morphology of the atherosclerotic plaque in Group I and Group II

	Group I (sympt. n = 30)			Group II (asympt. n = 40)		
Stenosis severity	Echolucent	Heterogeneous	Echogenic	Echolucent	Heterogeneous	Echogenic
< 60%	3 (10%)	4 (13.3%)	1 (3.3%)	–	–	–
60 ≥ x < 80%	6 (20%)	5 (16.7%)	3 (10.0%)	2 (5%)	5 (12.5%)	7 (17.5%)
x ≥ 80%	1 (3.3%)	3 (10%)	4 (13.3%)	6 (15%)	9 (22,5%)	11 (27.5%)

Correlation of stenosis severity of echolucent plaque between Group I and Group II showed statistical significance with p-value 0.025, while statistical analysis of echogenic plaque showed none significance (*p*-value 0.352)

*Sympt* symptomatic individuals with carotid stenosis, *asympt* asymptomatic individuals with carotid stenosis

### Results of histological assessment

Histological assessment was performed to test the statistical significance of the quality of atherosclerotic plaque density interpretation using ultrasound. Ultrasound detected unstable plaques (patients with echolucent and heterogeneous carotid plaque) in 62.9% of patients, while histological assessment detected unstable plaques in 64.3% of patients. It indicated that ultrasound interpretation of the atherosclerotic plaque structure is highly informative and could be considered in assessing the risk of developing atherosclerotic complications.

### Results of vascular marker examination

Among vascular inflammatory markers, lipoprotein-associated phospholipase A2 (Lp-PLA2) showed a statistically significant difference (*P* < 0.01) in both the structure of atherosclerotic plaque and its clinical manifestations. In the patients studied, the concentration of Lp-PLA2was increased at 252.7–328.6 ng/ml in correlation with negative controls 205–240 ng/ml.

**Fig. 1:**
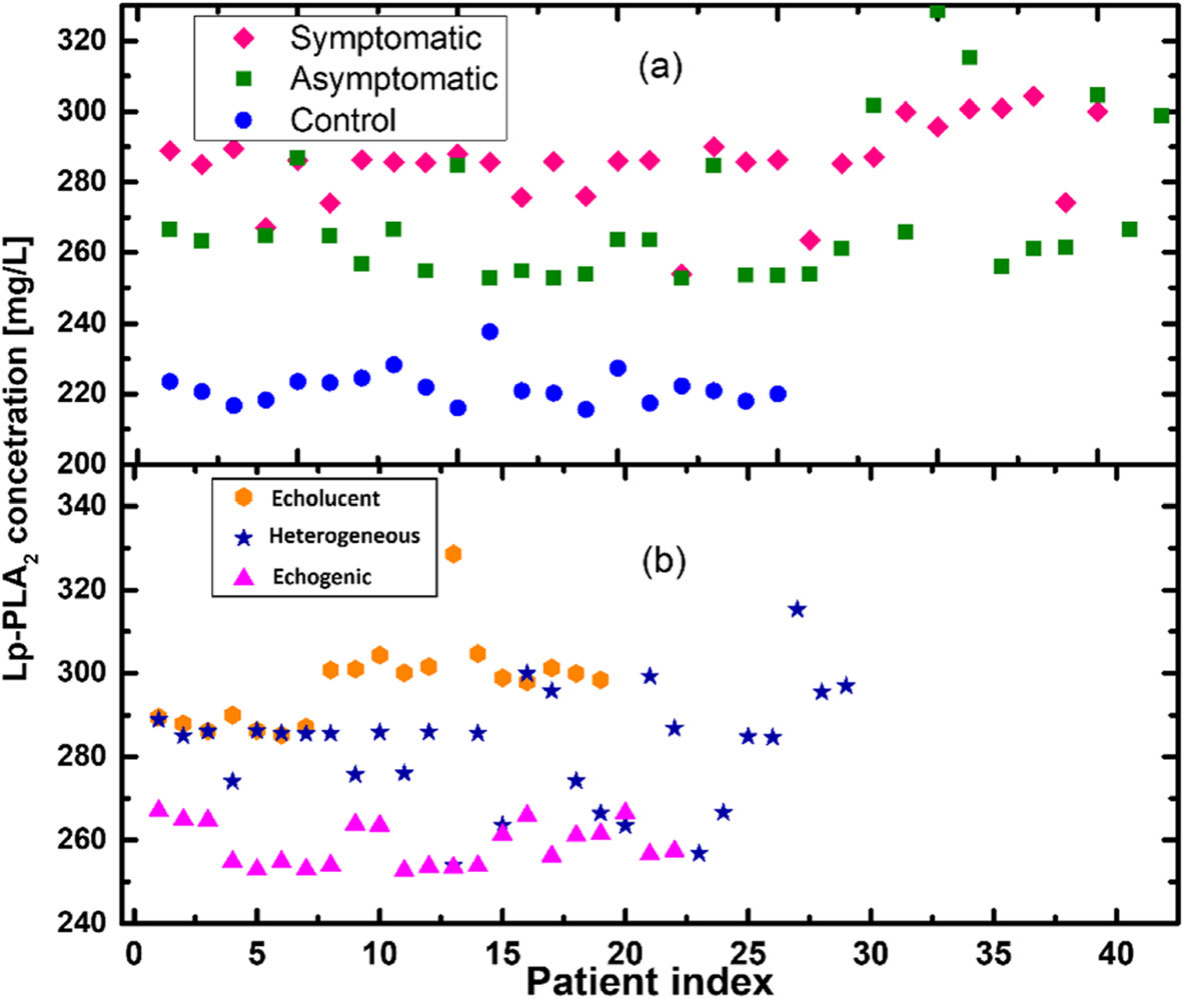
Changes in concentration of Lp-PLA_2_ according to symptomatic manifestations and plaque stability. (**a**) Concentration of Lp-PLA_2_ in symptomatic, asymptomatic patients and control group. Symptomatic = symptomatic individuals with carotid stenosis; Asymptomatic = asymptomatic individuals with carotid stenosis; Control = control group; (**b**) Concentration of Lp-PLA_2_ in patients with echolucent atherosclerotic plaque, heterogeneous atherosclerotic plaque and patients with echogenic atherosclerotic plaque; Echolucent = echolucent atherosclerotic plaque, Heterogeneous = heterogeneous atherosclerotic plaque, Echogenic = echogenic atherosclerotic plaque

When studying vascular inflammatory markers, the ratio of Lp-PLA2 concentration was compared between patients of Group I and those of Group II (symptomatic and asymptomatic ICA stenosis). The symptomatic group had a higher mean plasma level of Lp-PLA2 (285.30 ± 2.05 μg/l) than the asymptomatic group (274.35 ± 3.38 μg/l). The difference in the Lp-PLA2 levels between the symptomatic and asymptomatic groups (Fig. 1a) was significant (*p* < 0.05). No significant differences were noted in the plasma levels of Lp-PLA2 between men and women within the two groups.

On the other hand, the level of Lp-PLA2in 15% of asymptomatic patients with echolucent carotid plaque was higher than that in symptomatic patients with echogenic atherosclerotic plaque.

In addition, the dependence of Lp-PLA2 concentration from the structure of the atherosclerotic plaque was evaluated. While assessing the obtained results, a statistical significance (*p* < 0.05) between Lp-PLA2 concentration in patients with echolucent atherosclerotic plaque, heterogeneous atherosclerotic plaque and patients with echogenic atherosclerotic plaque was detected (Fig. 1b).

When assessing the results, a statistical significance was found between the increase in Lp-PLA2 concentration and the morphological structure of the atherosclerotic plaque (*p* < 0.001).

**Fig. 2:**
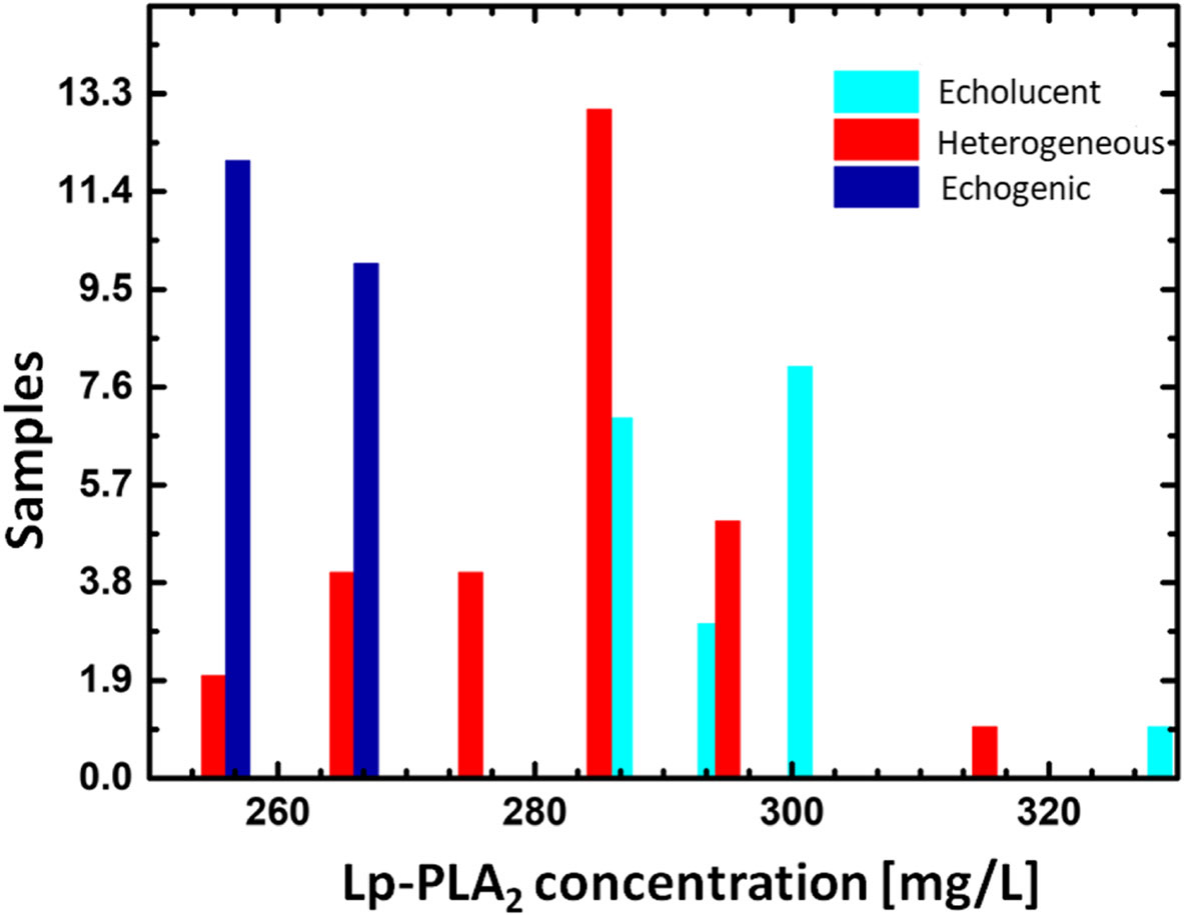
Histograms for echolucent (cyan), heterogeneous (red), and echogenic (blue) carotic plaques. The results are statistically significant at *p*<0.001 (the Pearson correlation coefficient, MINITAB Inc., Coventry, United Kingdom)

Histograms in Fig. 2 indicate a number of patients with a different type of hardness based on Lp-PLA2 concentration. Noteworthy, the peaks and tails of histograms overlap. That means that we cannot separate or identify patients using only Lp-PLA2 concentration level. One could notice from Fig. 1a that symptomatic individuals with carotid stenosis have Lp-PLA2 > 250 mg/L. In the current paper, we propose to take into account additional parameters such as Systolic to Diastolic Velocities Ratio (SDVR) of IVA and the type of plaque.

**Fig. 3:**
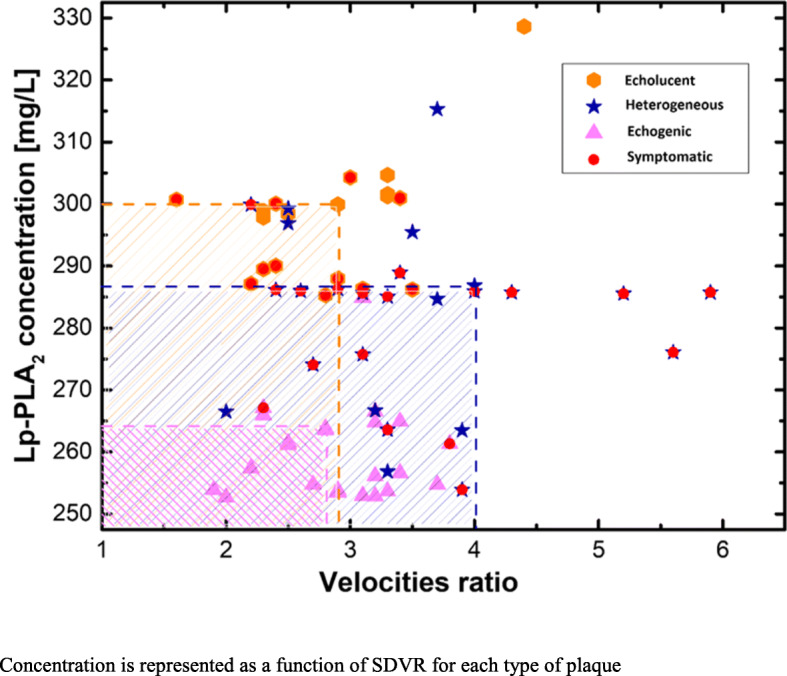
Lp-PLA2 concentration as a function of SDVR for each type of plaque. Concentration is represented as a function of SDVR for each type of plaque

In Fig. 3 Lp-PLA2 concentration is represented as a function of SDVR for each type of plaque. The red dots denote the symptomatic individuals with carotid stenosis inside the corresponding types of plaque. For the echolucent atherosclerotic plaque, red dots are spread almost homogenously. However, for the heterogeneous and echogenic atherosclerotic plaque, the red dots are located close to the edge of the point locations. The latter fact can be easily explained by the multiplication of two parameters Lp-LPA2 and SDVR. The geometrical outcome of the multiplication is the area of the corresponding rectangle. In Fig. 3, three rectangles are shown for one point belonging to each type of atherosclerotic plaque. Thus, the higher the value of the area, the higher the probability for a symptomatic individual with carotid stenosis. We propose the area, i.e., Lp-PLA2 * SDVR, as a modified parameter to predict future stroke attributed to carotid stenosis. Moreover, one can introduce the scaling factor (SF) for each type of plaque defining the high risk at 100. To satisfy the latter assumption, one must have SF = 5 for the echolucent atherosclerotic plaque, and SF = 10 and SF = 15 for heterogeneous and echogenic atherosclerotic plaques, correspondingly. The risk factor consequence can be calculated as follows:

$$ \mathrm{RF}=\mathrm{area}/\mathrm{SF} $$

We need to provide a more efficient way to determine patient status. Using an empiric equation, we are introducing the Risk index as the universal parameter to identify patient status.

**Fig. 4:**
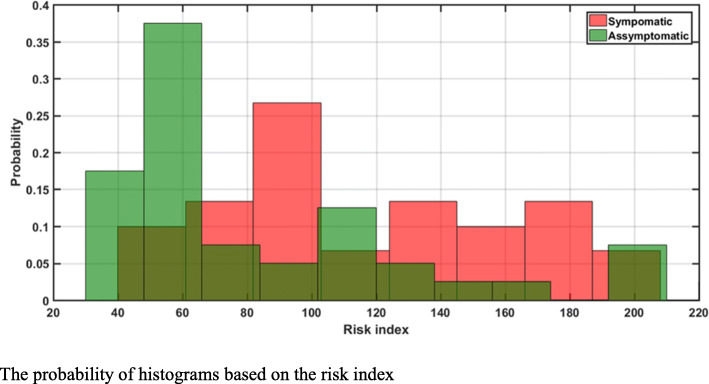
Determination of the risk of stroke development in symptomatic and asymptomatic patients. The probability of histograms based on the risk index

From Fig. 4, one can see the separate peaks for symptomatic and asymptomatic patients with probabilities 37% (asymptomatic) and 26% (symptomatic) which correspond to risk indices 60 and 90, respectively. Even though, the tails are overlapping more than the half asymptomatic patients have risk index < 70 while the majority of the symptomatic patients, 80% have risk index > 70. One can underline that the risk index is independent of gender, age of patients and depends only on well-defined and measured values such as Lp-LPA2 concentration and velocities ratio.

**Fig. 5:**
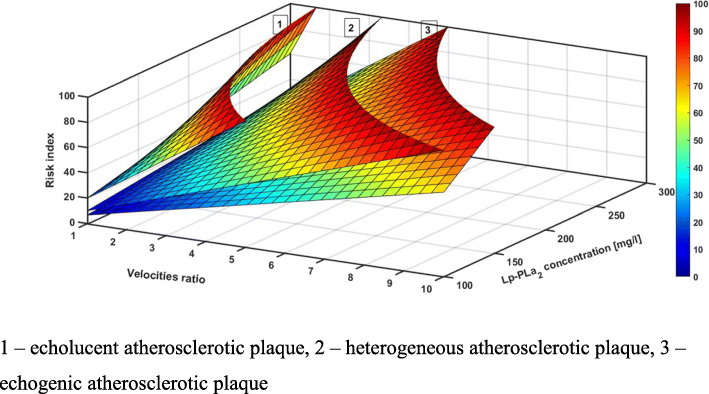
Ratio of ICA stenosis, atherosclerotic plaque structure and Lp-PLA_2_ concentration. 1 – echolucent atherosclerotic plaque, 2 – heterogeneous atherosclerotic plaque, 3 – echogenic atherosclerotic plaque

To assess the dominant risk factor for stroke, we analyzed the relationship between the degree of ICA stenosis, the morphological structure of the atherosclerotic plaque and the concentration of inflammatory markers in all the patients (Fig. 5). We deliberately compared symptomatic and asymptomatic individuals with carotid stenosis as symptomatic individuals, in our opinion, are crucial for determining the risk factor for stroke.

**Fig. 6:**
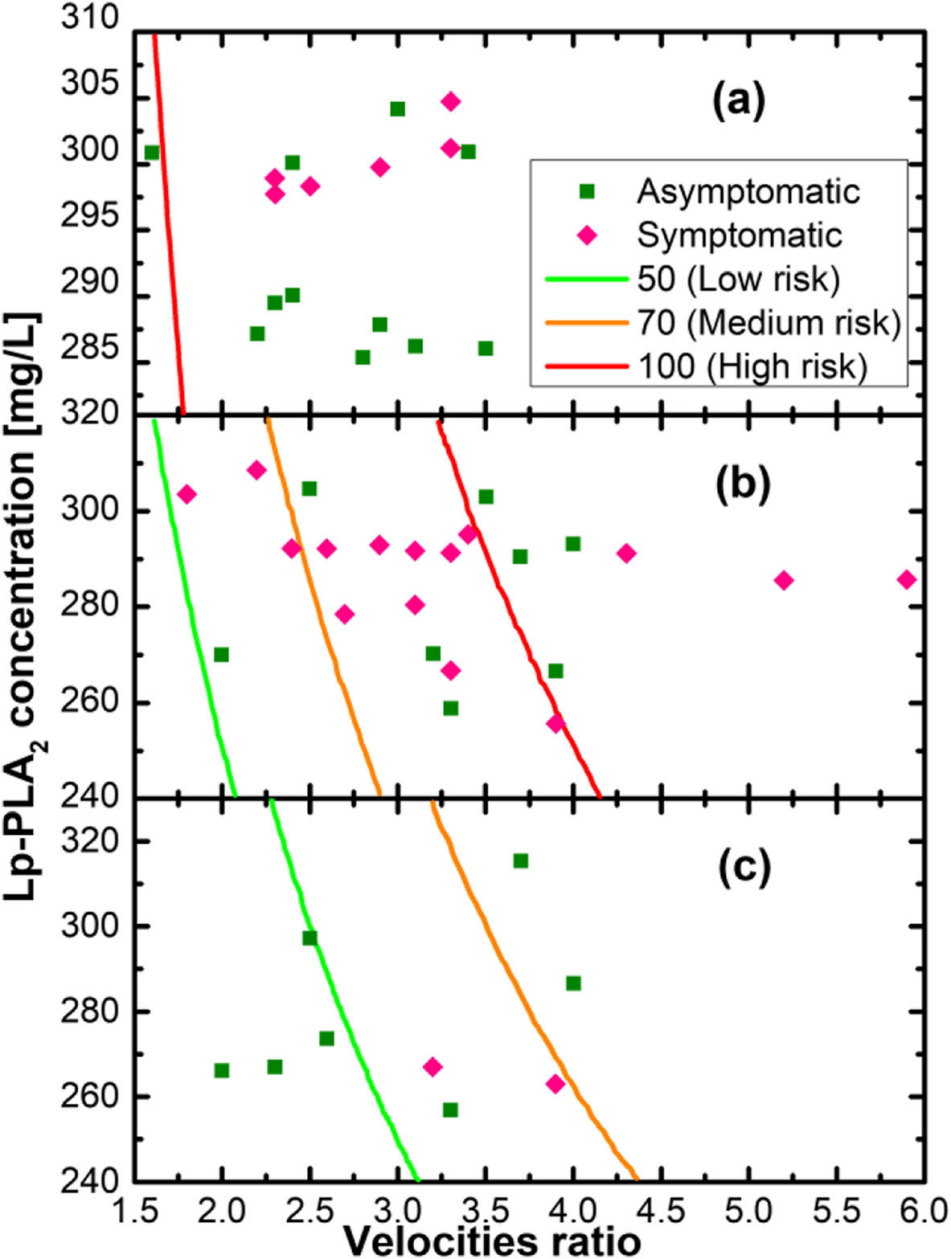
Correlation between concentration of Lp-PLA_2_ and velocities ratio according to plaque stability. (**a**) Risk of stroke development in patients with echolucent atherosclerotic plaque; (**b**) Risk of stroke development in patients with heterogeneous atherosclerotic plaque; (**c**) Risk of stroke development in patients with echogenic atherosclerotic plaque

According to the given empirical scale, all the patients with echolucent atherosclerotic plaque (both symptomatic and asymptomatic ones), ICA stenosis greater than 70% and Lp-PLA2 concentration of more than 285 mg/l had the risk of stroke development of > 100 points (Fig. 6a).

Among patients with heterogeneous atherosclerotic plaque, the risk of stroke development with > 100 points was observed in patients with hemodynamically significant ICA stenosis (> 80%) and Lp-PLA2 concentration of more than 285 mg/l. (Fig. 6b).

Patients with echogenic atherosclerotic plaque had low or medium risk of stroke development (Fig. 6c).

Based on the results obtained, a mathematical calculation of the major risk factors (the degree of stenosis, the morphological structure of the atherosclerotic plaque and the level of inflammatory marker concentration) has been proposed to calculate the risk index for developing of stroke in asymptomatic individuals with carotid stenosis.

The principle of mathematical calculation using the formula “Stroke-Stop” is the following: in patients with asymptomatic atherosclerotic disease of the carotid arteries, the concentration of Lp-PLA_2_ is determined using ELISA; both diastolic velocity and systolic velocity of blood flow within the ICA are measured using ultrasound thereby determining the degree of ICA stenosis; the structure of the atherosclerotic plaque is evaluated; and finally, the risk index for development of ischemic stroke using the proposed formula “Stroke-Stop” is calculated.

$$ \mathrm{Stroke}-\mathrm{Stop}"=\frac{\frac{\mathrm{systolic}\ \mathrm{velocity}\ \mathrm{of}\ \mathrm{the}\ \mathrm{ICA}}{\mathrm{diastolic}\ \mathrm{velocity}\ \mathrm{of}\ \mathrm{the}\ \mathrm{ICA}}\ast \mathrm{level}\ \mathrm{of}\ \left(\mathrm{Lp}-\mathrm{PLA}2\right)}{\mathrm{coefficient}\ \mathrm{of}\ \mathrm{atherosclerotic}\ \mathrm{plaque}\ \mathrm{density}\ } $$

The numerator represents the ratio of ICA systolic velocity to ICA diastolic velocity multiplied by the indicator of Lp-PLA2 concentration, and the denominator represents the density coefficient of “5” in echolucent atherosclerotic plaque, the density coefficient of “10” in heterogeneous atherosclerotic plaque and the density coefficient of “15” in echogenic atherosclerotic plaque. If the indicator is 50–70 points, the index of stroke risk is low, if the indicator is from 70 to 100 points, the index of stroke risk is medium, and if the indicator is more than 100 points – the index of stroke risk is high.

The use of the coefficient does not affect changes in the results since it is used as an identifier of the atherosclerotic plaque structure in all the patients. The results of calculating the risk of developing ischemic stroke by the formula “Stroke-Stop” are presented in Table 3.

**Table 3:** Calculation of the risk of stroke development by the “Stroke-Stop” formula

№	Index “Stroke –Stop”	Lp-PLA_**2**_	Plaque	Stenosis	Sympt Patients	Asympt patients
**Minimal Risk**						
1	58	252.9	Echogenic	70		+
2	59	256.1	Echogenic	70		+
3	62	265.9	Echogenic	70		+
4	64	261.1	Echogenic	75		+
5	68	268.2	Echogenic	80		+
6	69	279.8	Echogenic	75		+
**Medium Risk**						
7	73	257.3	Echogenic	80		+
8	78	261.3	Echogenic	80		+
9	79	253.8	Echogenic	85		+
10	79	266.5	Echogenic	85		+
11	82	253.6	Echogenic	85		+
12	83	263.5	Echogenic	85		+
13	83	252.8	Echogenic	85		+
14	86	253.9	Echogenic	89		+
15	86	254.7	Echogenic	87		+
16	87	256.6	Echogenic	90		+
17	89	263.8	Echogenic	87		+
18	89	254.7	Heterogeneous	70		+
19	91	252.7	Echogenic	90		+
20	92	253.4	Echogenic	90		+
21	93	267.1	Echogenic	90		+
22	98	264.9	Heterogeneous	75		+
**High Risk**						
23	102	267.1	Echogenic	70	+	
24	102	276.1	Heterogeneous	65	+	
25	107	274.1	Heterogeneous	50	+	
26	107	285.9	Heterogeneous	60	+	
27	111	264.7	Heterogeneous	85		+
28	112	266.7	Heterogeneous	80		+
29	112	288.9	Heterogeneous	70	+	
30	115	256.8	Heterogeneous	85		+
31	115	285.3	Echolucent	55	+	
32	117	285.1	Heterogeneous	80	+	
33	117	286.1	Heterogeneous	50	+	
34	118	263.5	Heterogeneous	90		+
35	118	263.5	Heterogeneous	85	+	
36	119	266.5	Heterogeneous	90		+
37	119	253.9	Heterogeneous	90	+	
38	123	290.1	Echolucent	75	+	
39	125	284.7	Heterogeneous	95		+
40	126	315.3	Heterogeneous	70		+
41	127	286.3	Echolucent	70	+	
42	127	285.7	Heterogeneous	90	+	
43	129	296.9	Heterogeneous	70		+
44	132	275.7	Heterogeneous	90	+	
45	136	285.7	Heterogeneous	90	+	
46	137	274.2	Heterogeneous	85	+	
47	137	285.6	Heterogeneous	95	+	
48	139	286.3	Heterogeneous	90	+	
49	139	285.9	Heterogeneous	90	+	
50	142	284.9	Heterogeneous	95		+
51	143	285.2	Heterogeneous	95	+	
52	148	295.7	Heterogeneous	80	+	
53	157	295.5	Heterogeneous	95		+
54	164	300.1	Echolucent	60	+	
55	210	300.9	Echolucent	85	+	
56	216	299.9	Echolucent	90		+
57	219	297.8	Echolucent	75		+
58	229	328.6	Echolucent	75		+
59	238	287.1	Echolucent	85	+	
60	239	299.9	Echolucent	80	+	
61	259	287.9	Echolucent	85	+	
62	260	289.5	Echolucent	90	+	
63	268	298.9	Echolucent	80		+
64	270	300.7	Echolucent	85	+	
65	278	286.2	Echolucent	95	+	
66	279	301.2	Echolucent	90		+
67	284	298.4	Echolucent	90		+
68	285	304.7	Echolucent	90		+
69	292	304.4	Echolucent	95	+	
70	293	301.6	Echolucent	90		+

Dividing of the studied samples into groups with minimum, medium and high risk of stroke development after interpretation of “Stroke-Stop” formula

*Sympt* symptomatic individuals with carotid stenosis, *asympt* asymptomatic individuals with carotid stenosis

The results of calculating the risk of developing ischemic stroke by the formula “Stroke-Stop” presented in Table 2 clearly show the dependence and the influence of three risk factors (ICA stenosis, atherosclerotic plaque structure and Lp-PLA2 concentration) on stroke development.

**Fig. 7:**
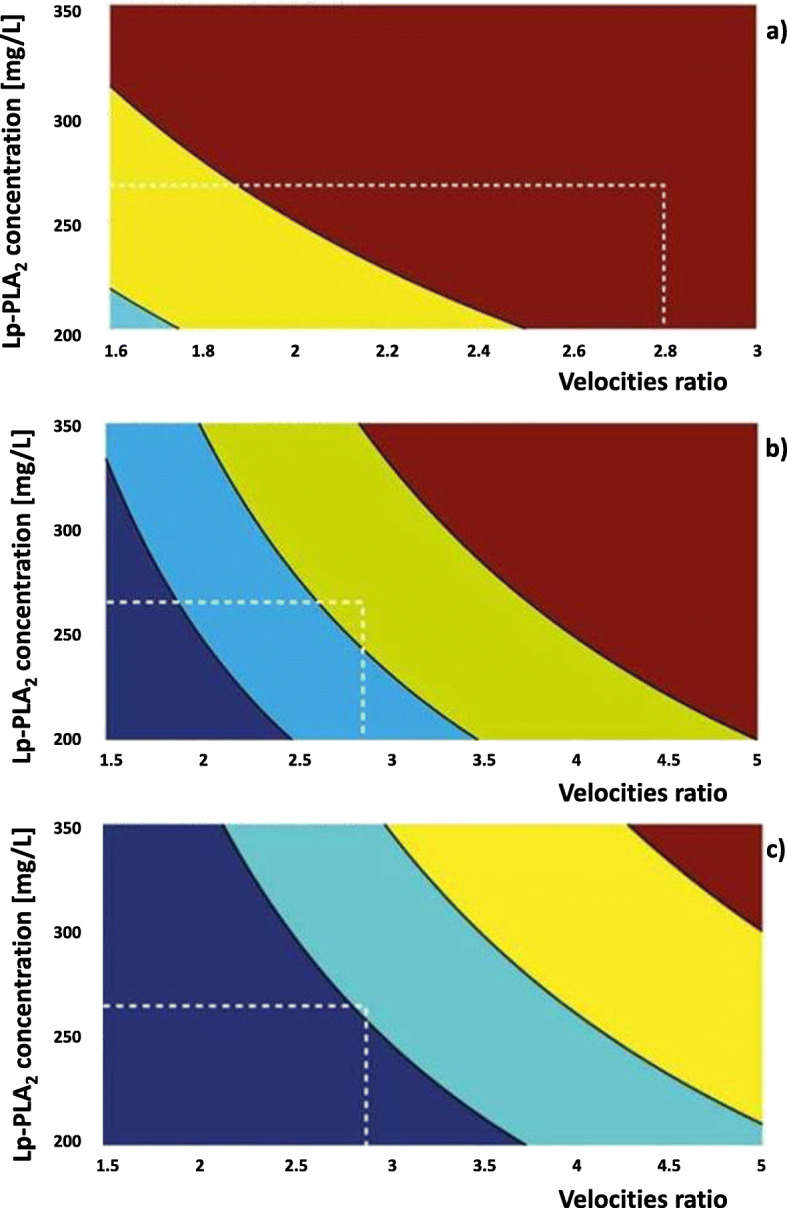
The graphical manifestation of risk of stroke calculated with “Stroke-Stop” formula. (**a**) Risk of stroke development in patients with echolucent atherosclerotic plaque; (**b**) Risk of stroke development in patients with heterogeneous atherosclerotic plaque; (**c**) Risk of stroke development in patients with echogenic atherosclerotic plaque

An alternative to the mathematical calculation of the risk of stroke development by the formula “Stroke-Stop” is its graphic display in the form of three diagrams, where each represents the structure of the atherosclerotic plaque (Fig. 7).

Red color points represent a high risk of stroke development; yellow color is used to indicate a medium risk of stroke development; blue color indicates a low risk of stroke development.

The diagrams (Fig. 7 a-c) show how the risk of stroke development in patients with ICA stenosis of 80% and Lp-PLA_2_ concentration at the level of 285 mg/l depends on the structure of the atherosclerotic plaque; Fig. 7a presents a high risk (echolucent atherosclerotic plaque), Fig. 7b presents a medium risk (heterogeneous atherosclerotic plaque); Fig. 7c presents a low risk (echogenic atherosclerotic plaque). The use of the diagram simplifies the process of determining the “Stroke-Stop” index as there is no need for mathematical calculations and the risk of stroke development is determined by the zone of contact between two indicators (ICA stenosis and Lp-PLA2 concentration) on the graphical chart where stroke risk threshold depends on the dominant risk factor, namely atherosclerotic plaque structure.

## Discussion

It was established that the carotid endarterectomy is the effective treatment to reduce the risk of subsequent stroke in symptomatic individuals with carotid stenosis [9]. Moreover, the risk-benefit ratio prefers surgery for about 50% symptomatic stenosis. In correlation with symptomatic stenosis, it is the unusual behavior for the asymptomatic lesions [13]. Therefore, the stenosis degree stand-alone might not be sufficient to predict the risk of a stroke. Clearly, additional markers are required to characterize more precisely the patients who would benefit the most from a surgery. Results of ACSRS study shows that specific baseline clinical characteristics and ultrasonic plaque features after image normalization can independently predict future ipsilateral cerebrovascular events [33–35].

The development of new diagnostic technologies is contributing to the transition to a multi-parameter systematic model that allows the formation of a personalized approach to the diagnosis and prevention of stroke.

The main criteria, evaluated in our study, were the degree of ICA stenosis, the morphological structure of the atherosclerotic plaque (determined by ultrasound) and the level of Lp-PLA2 concentration.

We propose to further study unique biomarker Lp-PLA2 – an enzyme produced by inflammatory cells and hydrolyzes oxidized phospholipids in LDL, which in connection with individual patient plague stability may lead to earlier detection of atherosclerosis progression / manifestation. Lipoprotein-associated phospholipase A2 is also known as platelet-activating factor acetylhydrolase (PAF-AH). In the blood it is mainly connected with low density lypoprotein (LDL, near 80%) and only less than 20% of this enzyme is associated with high density lypoprotein (HDL) [25].

Mannheim et al. concluded in their study that symptomatic carotid artery plaques are characterized by increased levels of Lp-PLA2 and its product Lysophosphatidylcholine in correlation with markers of tissue oxidative stress, inflammation, and instability. These findings strongly support a role for Lp-PLA2 in the pathophysiology and clinical presentation of cerebrovascular disease [22].

The combination of three factors – stenosis, ulceration of the atherosclerotic plaque and the inflammatory process within it – is one of the leading mechanisms of embologenicity as well as the development of stroke [30, 36]. This mechanism is not taken into consideration by the classical approach to determining the indications for carotid endarterectomy where the main criterion is the degree of ICA stenosis (50% in symptomatic patients and 60–99% in asymptomatic patients).

Alongside with the degree of stenosis, plaque morphology can provide crucial information to predict the stroke risk. The recent ultrasound studies showed a higher risk of cerebrovascular events for hypo- or anechogenic plaques compared to echogenic ones [31].

Among symptomatic patients, unstable carotid plaques were found in 76.6% of cases, while stable plaques were detected in 23.4% of cases only. The difference was statistically significant (*p* < 0.0001).

Unstable carotid plaques are associated with increased risk of stroke not only in symptomatic but also in asymptomatic patients. A meta-analysis of eight prospective studies, with a total of 7557 patients, observing patients with asymptomatic carotid stenosis found that patients with unstable, echolucent plaques had a 2.31-fold increased risk of stroke compared to patients with stable plaque based on ultrasound assessment [37].

Although, according to the study ACST-1, carotid plaque echolucency assessment offered no predictive value for stroke risk [38].

In our research, 25% of patients with asymptomatic ICA stenosis had unstable atherosclerotic plaque.

Experimental studies have shown a key role of inflammation in destabilization and rupture of the atherosclerotic plaque [15, 17, 39]. Specific vascular markers may serve as one of the criteria for assessing inflammation and destabilization of atherosclerotic plaque. In scientific journals, many articles on the comparison of different vascular markers were published [18, 19]. However, Lp-PLA2 is currently considered an independent biomarker for stroke, as well as coronary artery disease and peripheral arterial occlusive disease [21].

The JUPITER trial confirmed that patients with high Lp-PLA2 activity had more than twofold higher risk of developing cardiovascular events compared to those with low Lp-PLA2 activity [40].

Our study showed that patients with symptomatic stenosis of the internal carotid artery had significantly higher plasma levels of Lp-PLA2 compared to patients with asymptomatic stenosis.

When assessing the results, there was found a statistical significance between the increase in Lp-PLA2 concentration and the morphological structure of the atherosclerotic plaque.

Our results strongly confirm the role of Lp-PLA2 in the pathophysiology and clinical presentation of an unstable carotid plaque. Similar to our findings, some studies have reported the association of increased plasma levels of Lp-PLA2 in patients with unstable atherosclerotic plaque [33, 37, 41, 42].

The obtained results indicated that in patients with soft atherosclerotic plaque, the level of Lp-PLA2 concentration was statistically higher compared to patients with hard atherosclerotic plaque. In addition, in patients with soft atherosclerotic plaque, the level of Lp-PLA2 increased to the level of Lp-PLA2 concentration observed in symptomatic patients.

Therefore, we can state that soft atherosclerotic plaque, as well as an increased concentration of Lp-PLA2, is a risk factor for developing stroke.

The innovative approach proposed, clinically verified individually detected formula for determining the risk of stroke development. It considers three main risk factors: the degree of ICA stenosis, atherosclerotic plaque structure and Lp-PLA2 concentration.

According to the results of our study, the consideration of several factors increases the accuracy of calculating the risk of stroke development for particular individuals, which is the basis for risk stratification algorithms.

The use of the proposed method for mathematical calculation of the risk index for stroke development using the formula “Stroke-Stop” may serve as an auxiliary criterion at the stage of determining and selecting treatment tactics for patients with ICA stenosis greater than 70%, and finally to apply predictive and prognostic patient-specific treatment of atherosclerosis supporting the shift from reactive medicine to predictive, preventive, and personalized medicine.

In addition, thus proposed formula accompanied and accomplished with lipid individual profile can be applied alternatively to typical post symptomatic treatment of atheroslerosis. HDL-associated Lp-PLA2 may substantially contribute to the HDL antiatherogenic activity, and could be additionally applicable for the prediction of the efficacy of prescribed medication. Correspondingly, our Stop-Stroke formula is recommended for implementation for personalized clinical application of therapies. It is anticipated that, ultimately, this change in diagnosis and therapy will help in the future design and development of new, more selective and effective therapies for each individual patient.

## Conclusions

Hypothesis of “Stroke-Stop” formula for the determination of the risk of stroke development is proposed for patients with asymptomatic internal carotid artery (ICA) stenosis. This formula is based on a mathematical calculation of the major risk factors for stroke: the degree of ICA stenosis, the morphological structure of the atherosclerotic plaque (determined by ultrasound) and the level of lipoprotein-associated phospholipase A2 (Lp-PLA2) concentration. Based on calculations using “Stroke-Stop” formula, three main groups were generated: low (< 70 points), medium (70–100 points) and high (> 100 points) risk of stroke development. Hypothesis of “Stroke-Stop” formula is proposed for better selection of patients who should be indicated for surgical treatment, and will be evaluated in prospective study. In order to verify this hypothesis, we plan to do prospective study using “Stroke-Stop” formula for ipsilateral annual stroke rate in asymptomatic individuals with carotid stenosis who recieve conservative therapy.

## Data Availability

The datasets used and/or analyzed during the current study are available from the corresponding author on reasonable request.
